# Supplementary material for: Optimized design and in vivo application of optogenetically functionalized Drosophila dopamine receptors
Source: Nat Commun. 2023 Dec 19;14:8434. doi: 10.1038/s41467-023-43970-0 (PMC10730509; doi:10.1038/s41467-023-43970-0)
Supplement: Supplementary file 1 — Supplementary Information [file 41467_2023_43970_MOESM1_ESM.pdf]

Supplementary Information

Optimized design and in vivo application of optogenetically functionalized Drosophila dopamine receptors

Fangmin Zhou, Alexandra-Madelaine Tichy, Bibi Nusreen Imambocus, Shreyas Sakharwade, Francisco J. Rodriguez Jimenez, Marco González Martínez, Ishrat Jahan, Margarita Habib, Nina Wilhelmy, Vanessa Burre, Tatjana Lömker, Kathrin Sauter, Charlotte Helfrich-Förster, Jan Pielage, Ilona C. Grunwald Kadow, Harald Janovjak, Peter Soba

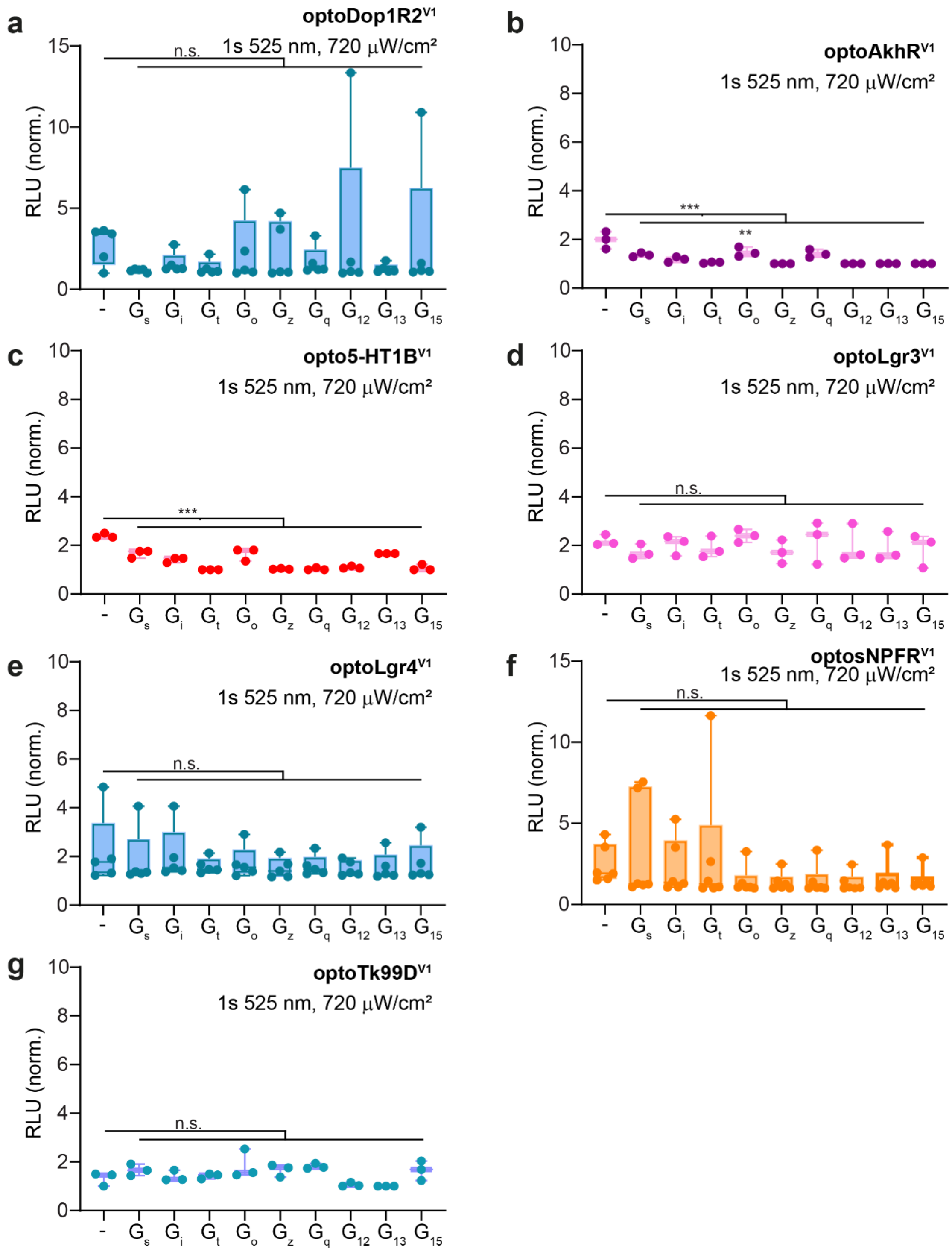

Supplementary Figure 1. Validation of optoXR^{V1} function in the G_{sx} assay.

a-g. Analysis of optoXR^{V1} function in live HEK293 cells using the G_{sx} assay to probe specific G protein activation. **a.** G protein coupling properties of optoDop1R2^{V1} after activation with light (1 s, 525 nm, 720 µW/cm^2). Maximum normalized responses are shown as relative light units (RLU, n=5 independent experiments, n.s. p>0.05). **b.** G protein coupling properties of optoAkhR^{V1} after activation with light (1s 525 nm, 720 µW/cm^2). Maximum normalized responses are shown as relative light units (RLU, n=3 independent experiments, **p<0.01, ***p<0.001, one-way ANOVA with Dunnett's *post-hoc* test). **c.** G protein coupling properties of opto5HT1B^{V1} after activation with light (1 s, 525 nm, 720 µW/cm^2). Maximum normalized responses are shown as relative light units (RLU, n=3 independent experiments, ***p<0.001, one-way ANOVA with Dunnett's *post-hoc* test). **d.** G protein coupling properties of optoLgr3

^{V1} after activation with light (1 s, 525 nm, 720 µW/cm^2). Maximum normalized responses are shown as relative light units (RLU, n=3 independent experiments, n.s. p>0.05, one-way ANOVA with Dunnett's *post-hoc* test). **e.** G protein coupling properties of optoLgr4^{V1} after activation with light (1s 525 nm, 720µW/cm^2). Maximum normalized responses are shown as relative light units (RLU, n=5, n.s. p>0.05, one-way ANOVA with Dunnett's *post-hoc* test). **f.** G protein coupling properties of optosNPFR^{V1} after activation with light (1 s, 525 nm, 720µW/cm^2). Maximum normalized responses are shown as relative light units (RLU, n=6 independent experiments, n.s. p>0.05, one-way ANOVA with Dunnett's *post-hoc* test). **g.** G protein coupling properties of optoTk99D^{V1} after activation with light (1 s, 525 nm, 720µW/cm^2). Maximum normalized responses are shown as relative light units (RLU, n=3 independent experiments, n.s. p>0.05, one-way ANOVA with Dunnett's *post-hoc* test). All boxplots depict 75th (top), median (central line) and 25th (bottom) percentile, whiskers depict 99th (top) and 1st (bottom) percentile. Source data and statistical details are provided as a Source Data file.

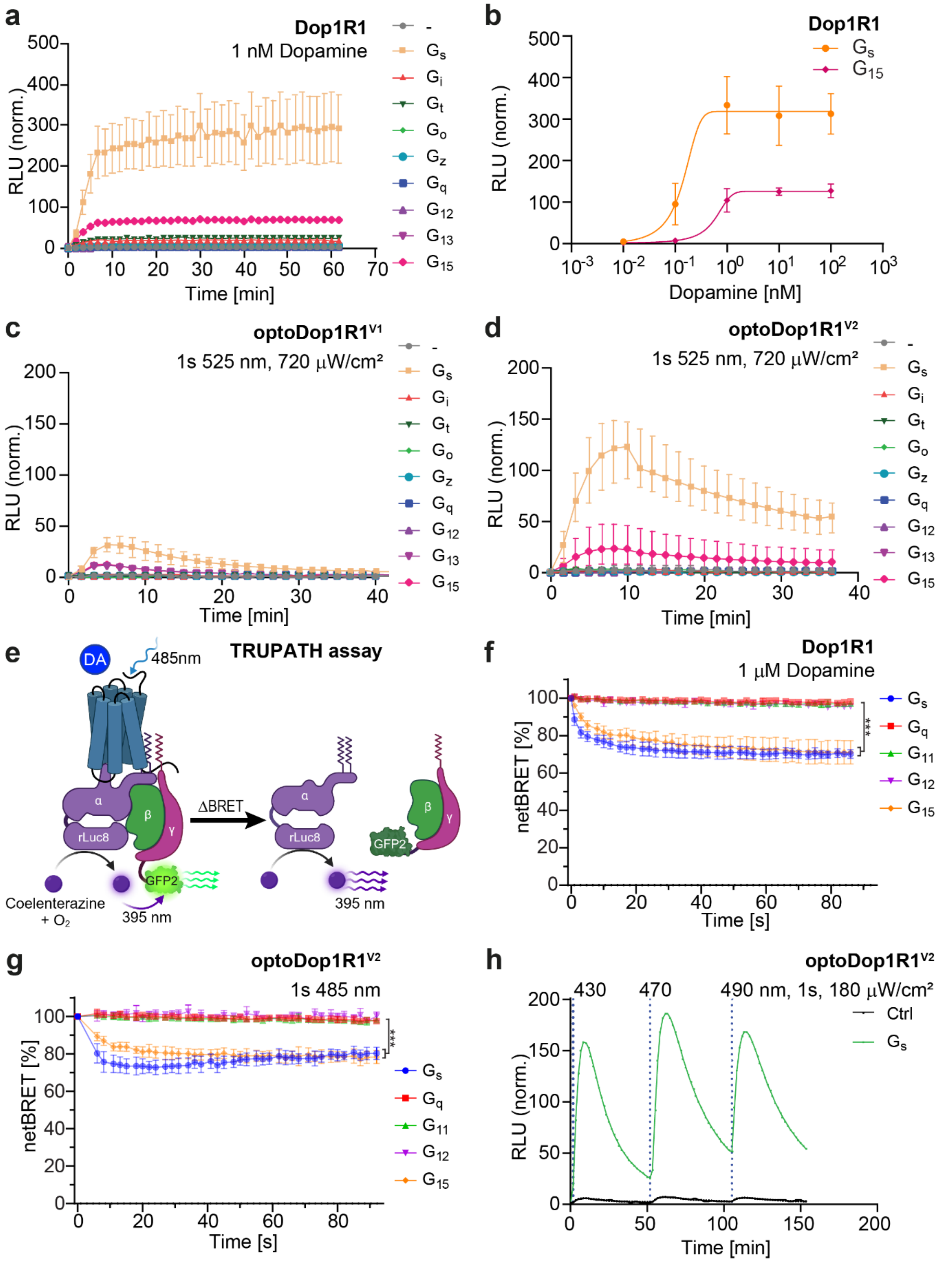

Supplementary Figure 2. Validation of optoDop1R1^{V2} function in G_{sx} and TRUPATH assays.

a-d. G protein coupling properties of *Drosophila* Dop1R1 and optoDop1R1 in the G_{sx} assay (shown as relative light units (RLU)). **a.** G protein coupling responses over time of *Drosophila* Dop1R1 with 1nM DA (mean ± SEM, n=4 independent experiments). **b.** DA concentration dependent maximum activation of G_s and G_{15} signaling of Dop1R1 (n=4 independent experiments). **c.** G protein coupling of optoDop1R1^{V1} after activation with light (1 s, 525 nm, 720 µW/cm^2). Normalized response kinetics are shown as relative light units (RLU, mean ±

SEM, n=7 independent experiments). **d.** G protein coupling properties of improved optoDop1R1^{V2} after activation with light (1s 525 nm, 720 μW/cm^2). Normalized response kinetics are shown as relative light units (RLU, mean ± SEM, n=7 independent experiments). **e.** Schematic of the TRUPATH assay. Bioluminescence resonance energy transfer (BRET) between Gα subunits fused to RLuc8 and Gγ subunits fused to GFP2 is diminished upon receptor activation and G protein subunit dissociation, resulting in lower BRET efficiency. Changes in the BRET emission ratio (netBRET: 515 nm/410 nm) represent G protein activation kinetics. Created with BioRender.com. **f.** Kinetic G protein coupling properties of *Drosophila* Dop1R1 after activation with 1μM DA assayed using TRUPATH (mean ± SEM, n=3 independent experiments, ***p<0.001, one-way ANOVA with Dunnett's *post-hoc* test). **g.** G protein coupling properties of optoDop1R1^{V2} after activation with light (1 s, 485 nm) using the TRUPATH assay. Normalized response kinetics are shown (mean ± SEM, n=4 independent experiments, ***p<0.001, one-way ANOVA with Dunnett's *post-hoc* test). **h.** Mean response of wavelength-dependent induction of G_s-mediated cAMP production after optoDop1R1^{V2} activation with light (1 s, 180 μW/cm^2, 430-490 nm, n=3 independent experiments). All boxplots depict 75th (top), median (central line) and 25th (bottom) percentile, whiskers depict 99th (top) and 1st (bottom) percentile. Source data and statistical details are provided as a Source Data file.

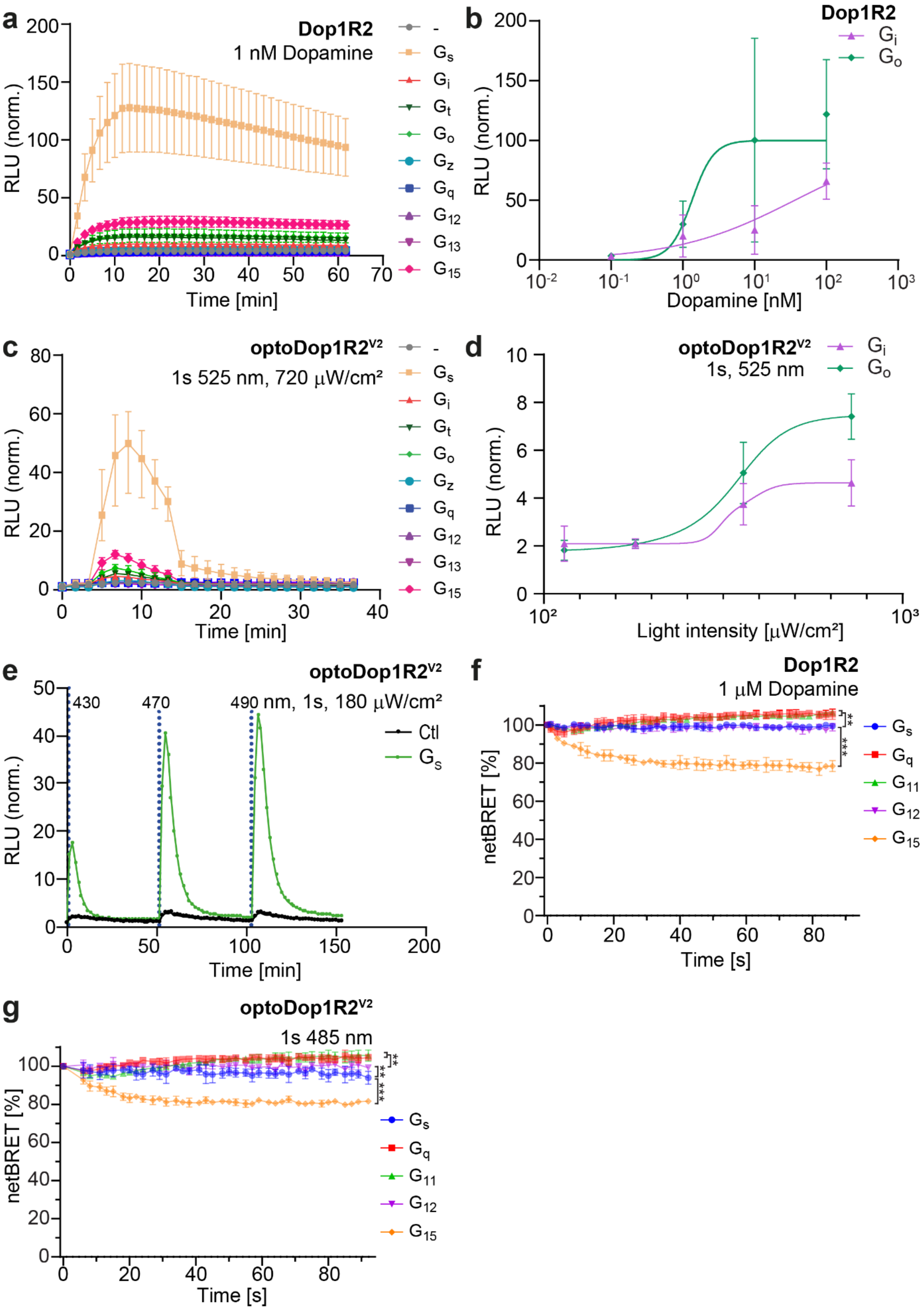

Supplementary Figure 3. Validation of optoDop1R2^{V2} function in G_{sx} and TRUPATH assays.

a-e. G protein coupling properties of *Drosophila* Dop1R2 and optoDop1R2^{V2} in the G_{sx} assay (shown as relative light units (RLU)). **a.** G protein coupling responses over time of Drosophila

Dop1R2 with 1nM DA (mean ± SEM, n=4 independent experiments). **b.** DA concentration dependent maximum activation of G_i and G_o signaling of Dop1R2 (mean ± SEM, 0.1/10 nM: n=3 independent experiments; 1.0/100 nM: n=4 independent experiments). **c.** G protein coupling responses over time of optoDop1R2^{V2} after activation with light (1s 525 nm, 720 μW/cm^2). Normalized response kinetics are shown (mean ± SEM, n=4 independent experiments). **d.** Light intensity-dependent maximum of G_i and G_o signaling induced by optoDop1R2^{V2} (1 s, 525 nm, n=4 independent experiments). **e.** Wavelength-dependent induction of G_s-mediated cAMP production after optoDop1R2^{V2} activation with light (1s 180 μW/cm^2, 430-490 nm, n=3 independent experiments). **f.** Kinetic G protein coupling properties of wild type Dop1R2 after activation with 1μM DA assayed with TRUPATH (mean ± SEM, n=3 independent experiments, one-way ANOVA with Dunnett's *post-hoc* test). **g.** G protein coupling properties of optoDop1R2^{V2} after activation with light (1 s, 485 nm) using TRUPATH. Normalized response kinetics are shown (mean ± SEM, n=4 independent experiments, one-way ANOVA with Dunnett's *post-hoc* test). All boxplots depict 75th (top), median (central line) and 25th (bottom) percentile, whiskers depict 99th (top) and 1st (bottom) percentile. Source data and statistical details are provided as a Source Data file.

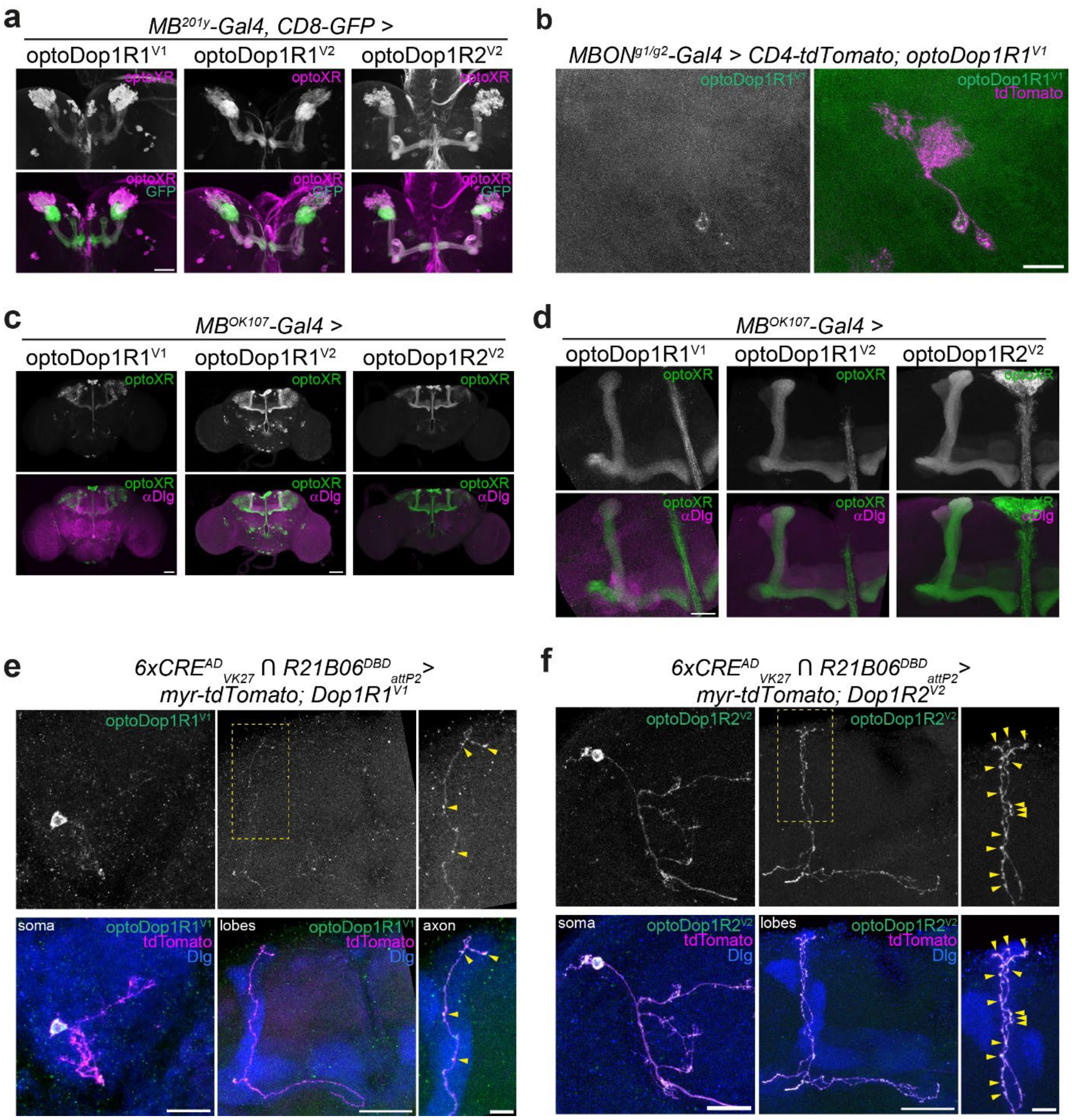

Supplementary Figure 4. *In vivo* localization of optoDopRs.

a. Overview of immunolabeled optoDopR expression in the larval mushroom body (*201y-Gal4, CD8-GFP,* scale bars: 50 μm). **b.** Single cell expression of immunolabeled optoDop1R1^{V1} in larval MBONs co-labeled with membrane bound CD4-tdTomato (*MBON$^{g1/g2}$-Gal4, CD4-tdTomato*). Scale bar 20 µm. Expression was mostly detected in the soma, with low expression in the axon and dendrites. **c.** Immunolabeling of optoDopRs in the adult mushroom body (all KCs, *OK107-Gal4, myr-tdTomato*) co-labeled with anti-Dlg marking the MB (scale bars: 50 μm). **d.** Enlarged view of optoDopR expression in the adult mushroom body (all KCs, *OK107-Gal4, myr-tdTomato*) co-labeled with anti-Dlg marking the MB (scale bars: 25 μm). **e.** Single cell immunolabeling of optoDop1R1^{V1} expressed in an adult mushroom body KC together with membrane-bound tdTomato and Dlg outlining the MB (scale bar: 10, 20, 5 µm). Prominent labeling is only seen in the KC soma with low dendritic and axonal signal. Arrowheads indicate axonal varicosities. **f.** Single cell expression of optoDop1R2^{V2} in the adult mushroom body showing a KC labeled with membrane bound tdTomato and immunostained for opto Dop1R2^{V2} and Dlg outlining the MB (scale bar: 10, 20, 5 µm). Prominent axonal and somatodendritic

localization can be detected, with arrowheads indicating axonal varicosities. All panels show representative images from at least two independent experiments with multiple samples.

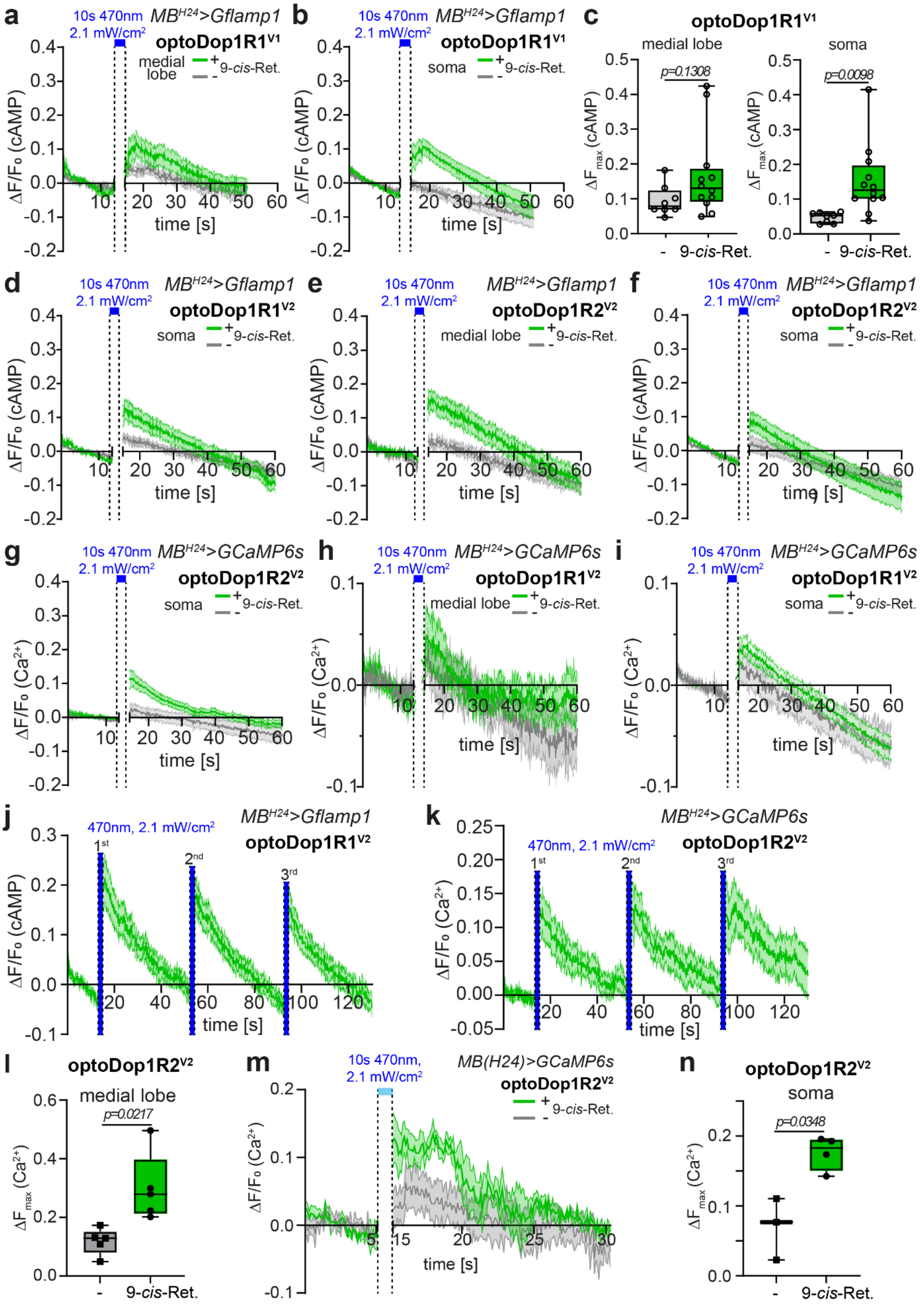

Supplementary Figure 5. *In vivo* validation of optoDopR activity.

a-c. cAMP imaging in the larval mushroom body using Gflamp1 and optoDop1R1[V1] expression with and without 9-*cis*-Retinal feeding. Responses in the medial lobe (**a**) and soma (**b**) after 10s blue light illumination are shown over time. Maximum cAMP responses in the medial lobe

and soma (**c**) after optoDop1R1^{V1} activation (n=8,12 biologically independent samples, two-tailed unpaired Student's *t*-test). **d.** cAMP imaging in the larval mushroom body using Gflamp1 and optoDop1R1^{V2} expression (*H24-Gal4>G-Flamp1, optoDop1R1^{V2}*, 10s 470 nm, n=11, 15). Responses in the soma after 10s blue light illumination are shown over time. **e-f.** cAMP imaging in the larval mushroom body using Gflamp1 and optoDop1R2^{V2} expression with and without 9-*cis*-Retinal feeding (10s 470 nm, n=7,8 biologically independent samples). Responses in the medial lobe (**e**) and soma (**f**) after 10s blue light illumination are shown over time. **g-i.** Calcium imaging in the mushroom body using GCaMP6s and optoDop1R2^{V2} or optoDop1R1^{V2} expression in isolated larval brains (10s 470 nm, optoDop1R2^{V2}: n=11,7 biologically independent samples; optoDop1R1^{V2}: n=8,8 biologically independent samples). Responses in the soma upon optoDop1R2^{V2} activation (**g**), and for optoDop1R1^{V2} activation in the medial lobe (**h**) and soma (**i**) are shown over time. **j-k.** cAMP or calcium imaging in the larval mushroom body in isolated brains with repeated light activation of optoDopRs (each light pulse: 10 s, 470 nm). Medial lobe cAMP responses upon optoDop1R1^{V2} activation (n=10 biologically independent samples) (**j**) and calcium responses upon optoDop1R2^{V2} activation (n=6 biologically independent samples) (**k**) are shown over time. **l-n.** *In vivo* calcium imaging in intact larvae using GCaMP6s and optoDop1R2^{V2} expressed in the larval mushroom body (*H24-Gal4>GCaMP6s, optoDop1R2^{V2}*). Maximum calcium responses in the MB medial lobe after light-induced activation of optoDop1R2^{V2} with or without 9-*cis*-retinal feeding (10s 470 nm, n=5,5 animals, two-tailed unpaired Student's *t*-test) (**l**). Calcium responses in KC somata with or without 9-*cis*-retinal over time (**m**) and maximum responses (**n**) after light-induced activation of optoDop1R2^{V2} (10s 470 nm, n=3, 4 animals, two-tailed unpaired Student's *t*-test).

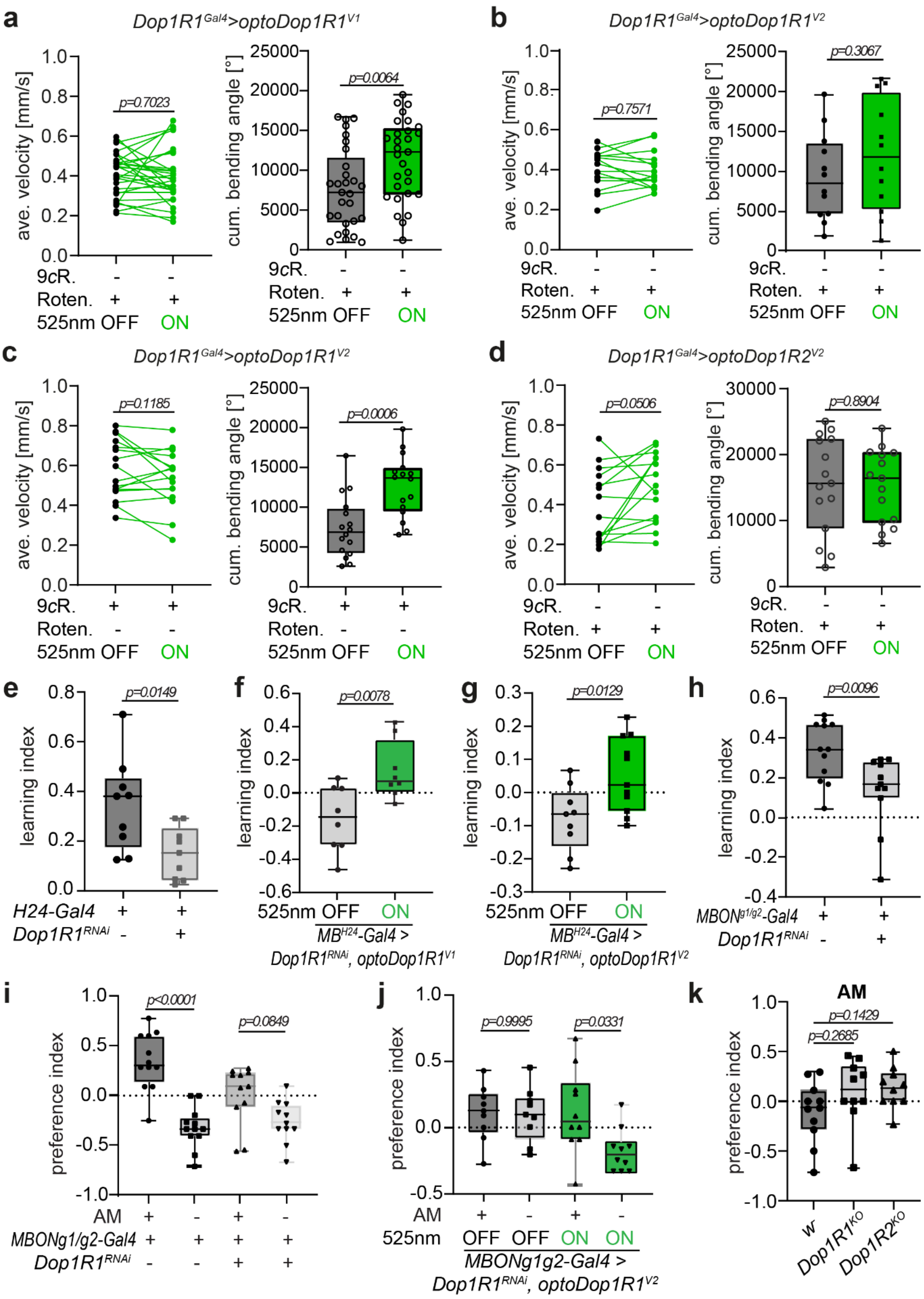

Supplementary Figure 6. Functional validation of optoDopRs in Drosophila larvae *in vivo.*

a-b. Average velocity and bending angles of Rotenone-fed animals expressing optoDop1R1^{V1}(**a**) or optoDop1R1^{V2} (**b**) in an endogenous Dop1R1-like pattern without 9-*cis*-

Retinal feeding. Animals were tracked without light for 1min and with 525 nm light illumination for 1 min. Average velocity (left) or cumulative bending angles (right) in the dark (OFF) and during light activation (ON) are shown (optoDop1R1^{V1}: n=29, 29 animals, optoDop1R1^{V2}: n=12,12 animals, two-tailed paired Student's *t*-test). **c.** Average velocity and bending angles of 9-*cis*-Retinal fed larvae without Rotenone treatment expressing optoDop1R1^{V2} in an endogenous Dop1R1-like pattern. Larvae were tracked without light for 1min and with 525 nm light illumination for 1 min. Average velocity (left) or cumulative bending angles (right) in the dark and during light activation are shown (n=16,16, ** p<0.01, paired Student's *t*-test). **d.** Average velocity and bending angles of Rotenone-fed animals expressing optoDop1R2^{V2} in an endogenous Dop1R1-like pattern without 9-*cis*-Retinal feeding. Larvae were tracked without light for 1min and with 525 nm light illumination for 1 min. Average velocity (left) or cumulative bending angles (right) in the dark and during light activation are shown (n=15,15, n.s. p>0.05, two-tailed paired Student's t-test). **e.** Larval learning after fructose-odor training is impaired upon Dop1R1^{RNAi} expression in the MB (n=9, 9 independent experiments, two-tailed unpaired Student's *t*-test). **f.** Dop1R1-dependent single odor-fructose learning in larvae. Animals expressing optoDop1R1^{V1} and Dop1R1^{RNAi} in KCs were trained using fructose-odor learning (3x3min) with or without light activation during fructose exposure (3 min 525 nm, 720 μW/cm^2). Learning index of 9-*cis*-Retinal fed animals with and without light activation during training is shown (n=8, 8 independent experiments, unpaired two-tailed Student's *t*-test). **g.** Single odor-fructose learning in larvae expressing optoDop1R1^{V2} and Dop1R1^{RNAi} in KCs. Fructose-odor learning (3x3min) with or without light activation during fructose exposure (3 min 525 nm, 720 μW/cm^2). Learning index of 9-*cis*-retinal fed animals with and without light activation during training is shown (n=9, 11 independent experiments, two-tailed unpaired Student's *t*-test). **h.** Larval learning after fructose-odor training is impaired upon Dop1R1^{RNAi} expression in MBON$^{g1/g2}$ (n=12, 11 independent experiments, two-tailed unpaired Student's *t*-test). **i** Fructose reward-dependent induction of odor preference (AM or blank) for Dop1R1-dependent data from (**h**) (n=12, 11 independent experiments, One-way ANOVA with Tukey's *post-hoc* test). **j.** Fructose and optoDop1R1^{V2}-dependent induction of odor preference (amylacetate (AM) or blank) with (green bars) or without (gray bars) light illumination during fructose pairing in Dop1R1^{RNAi} larvae (n=9, 9 independent experiments, One-way ANOVA with Tukey's *post-hoc* test, data from Fig. 5e). **k.** Innate preference index for AM in control (w$^-$), *Dop1R1^{KO}* and *Dop1R2^{KO}* 3rd instar larvae (n=11, 10, 9 independent experiments, One-way ANOVA with Tukey's *post-hoc* test). All boxplots depict 75th (top), median (central line) and 25th (bottom) percentile, whiskers depict 99th (top) and 1st (bottom) percentile. Source data and statistical details are provided as a Source Data file.

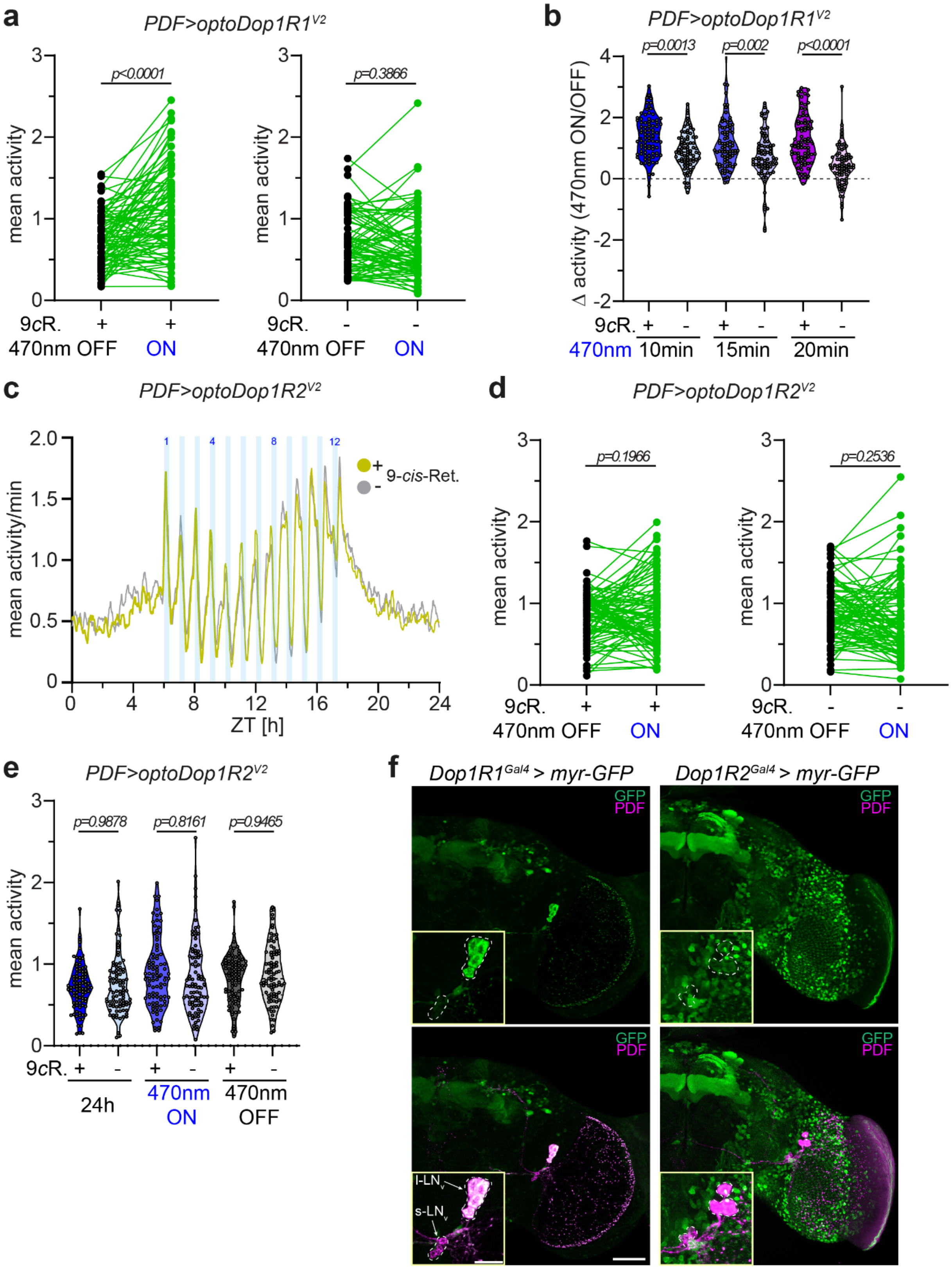

Supplementary Figure 7. Cell type-specific function of Dop1R1 activity in blue light induced arousal.

a. Activity difference of flies expressing optoDop1R1^{V2} in PDF neurons with (left) and without 9-*cis*-Retinal (9cR) feeding (right) before and during blue light pulse exposure (24h activity data from Fig. 6g, n=83,77 animals, two-tailed paired *t*-test). **b.** Activity difference of flies expressing optoDop1R1^{V2} in PDF neurons (with and without 9-*cis*-Retinal feeding) during light on times using different duration of blue light pulse exposure (1/h, 10, 15 or 20min,

n=83,77 animals, one-way ANOVA with Tukey's *post-hoc* test). **c.** Mean activity during 24h monitoring in flies expressing optoDop1R2^{V2} in pdf neurons with and without 9cR feeding (n=90 animals). Blue light pulses (12x 20min, 1/h) during daytime increase fly activity independently of optoDop1R1^{V2} activation. **d.** Activity difference of flies expressing optoDop1R2^{V2} in PDF neurons with (left) and without 9-*cis*-Retinal (9cR) feeding (right) before and during blue light pulse exposure (24h activity data from Fig. S6e, (n=90 animals, two-tailed paired *t*-test). **e.** Mean activity of *pdf>optoDop1R2*^{V2} -expressing flies during the entire 24h, all light on and light off phases (n=90 animals, one-way ANOVA with Tukey's *post-hoc* test). **f.** myristoylated (myr-)GFP reporter expression using Dop1R1 (left panel) or Dop1R2 (right panel) knock-in Gal4 lines together with immunolabeling of PDF-expressing s-LN_vs and l-LN_Vs (somata are indicated by dotted lines). Note that Dop1R1 reporter expression is specific for l-LN_Vs, while Dop1R2 reporter expression is weak in all LN_vs. Scale bar: 50μm, inset 10μm. All violin plots with single data points depict data distribution, dotted lines depict 75th (top) and 25th (bottom) percentile, solid central line the median. Source data and statistical details are provided as a Source Data file.

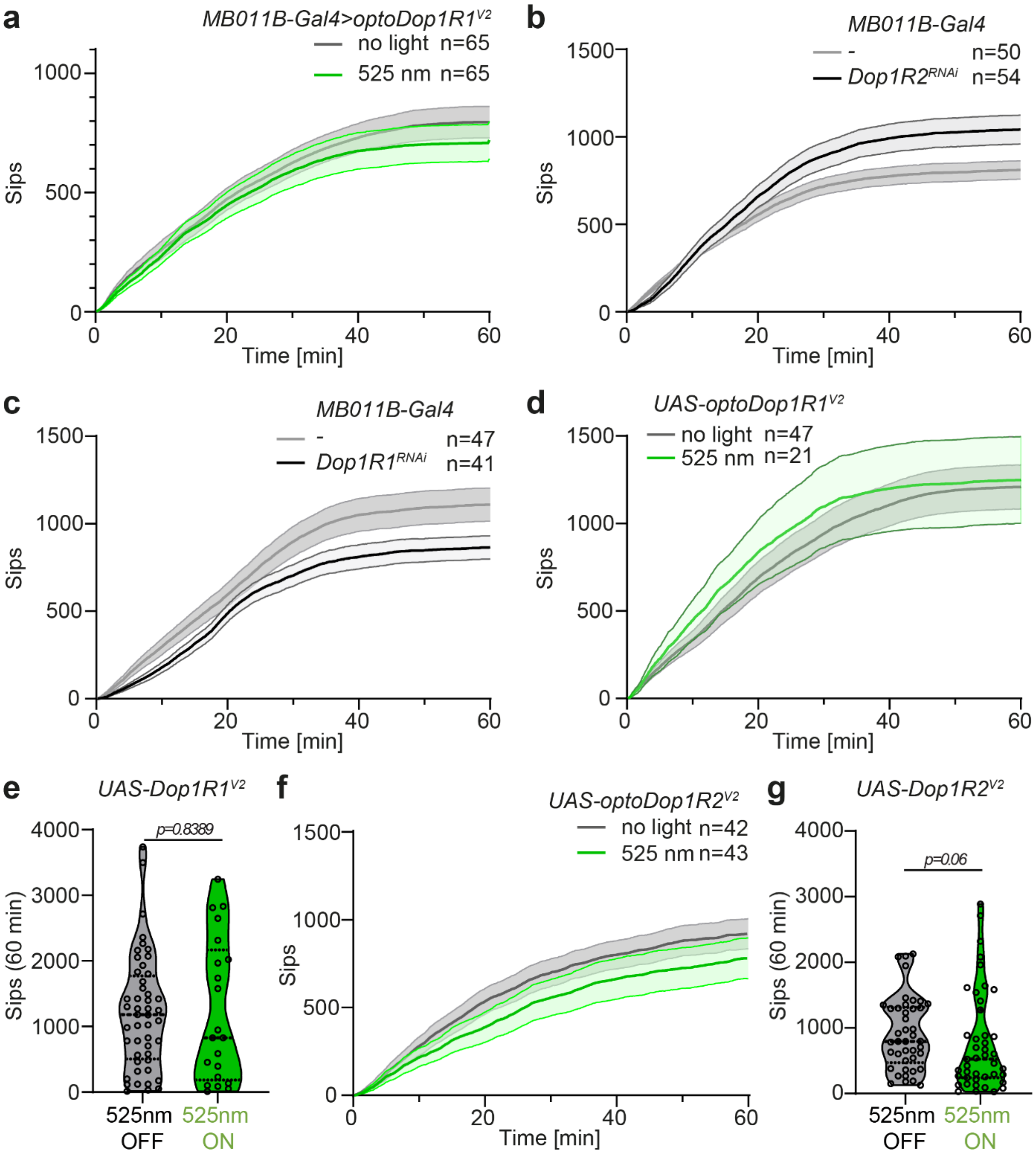

Supplementary Figure 8: Cell type-specific function of Dop1R2 activity in satiety.
a. Cumulative sips over time in flies expressing optoDop1R1^{V2} with *MB011B-Gal4* with or without light stimulation (mean ± SEM, n=65,65 animals). **b.** Cumulative sips over time in flies expressing Dop1R2^{RNAi} with *MB011B-Gal4* compared to control (mean ± SEM, n=50,54 animals). **c.** Cumulative sips over time in flies expressing Dop1R1^{RNAi} with *MB011B-Gal4* (mean ± SEM, n=47,41 animals). **d.** Cumulative sips over time in optoDop1R1^{V2} transgenes without Gal4 expression and without or with light stimulation (mean ± SEM, n=48,21 animals). **e.** Total sips at 60min for optoDop1R1^{V2} transgenes without Gal4 expression and without or with light stimulation (n=48,21 animals, two-tailed Mann-Whitney test). **f.** Cumulative sips over time in optoDop1R2^{V2} transgenes without Gal4 expression and without or with light stimulation (mean ± SEM, n=42,43 animals). **g.** Total sips at 60min for optoDop1R2^{V2} transgenes without Gal4 expression and without or with light stimulation (n=42,43 animals, two-tailed Mann-Whitney test). All violin plots with single data points depict

data distribution, dotted lines depict 75th (top) and 25th (bottom) percentile, solid central line the median. Source data and statistical details are provided as a Source Data file.

Supplementary Table 1. Previous optoXRs and their *in vivo* applications. Only optoXRs that have been applied *in vivo* are listed here. Abbreviations: Rho: bovine Rhodopsin, OPN4: melanopsin

**Chimeric receptor**	**Original reference**	***In vivo* applications**	**Cell type-specificity/rescue of endogenous receptor function**
Rho:β_2AR	[1]	- virus-mediated overexpression in mouse N. accumbens neurons [2]<br>- virus-mediated overexpression in mouse basolateral amygdala, promoting anxiety-like behavior [3,4]	partial/no<br>partial/no
Rho:α_1AR	[2]	- virus-mediated overexpression in mouse N. accumbens neurons, reward-related preference behavior<br>- virus-mediated overexpression in mouse CA1 astrocytes, memory acquisition[5]<br>- transgenic overexpression in mouse cortical astrocytes, remote memory acquisition [6]<br>- virus-mediated overexpression in mouse astrocytes in slices, electrophysiology [7]	partial/no<br>partial/no<br>partial/no<br>partial/no
Rho:μOR	[8]	- virus-mediated overexpression in mouse dorsal root ganglion neurons, preference/aversion behavior [9]<br>- Penk-Cre dependent virus-mediated overexpression in dorsal raphe nucleus subset neurons, restoration of consumption behavior [10]	partial/no<br>yes/yes
Rho:DRD1	[11]	- DRD1-Cre dependent virus-mediated overexpression in mouse N. accumbens; activation of medium spiny neurons to increase social interaction	yes/no
Rho:CXCR4	[12]	- virus-mediated overexpression in mouse, T-cell recruitment	yes/no
Rho:A_{2A}R	[13]	- virus-mediated overexpression in mouse hippocampus and N. accumbens, spatial memory performance and locomotor activity<br>- adora2a-cre dependent virus-mediated overexpression in mouse striatopallidal neurons, goal-directed behavior [14]	partial/no<br>yes/no
OPN4:$mGluR_6$	[15]	- virus-mediated overexpression in retinal ganglion cells, restoration of visually guided behavior<br>- virus-mediated overexpression in bipolar cells, restoration of visually guided behavior [16]	yes/partial (degeneration model)<br>yes/yes (degeneration model)
Rho:Fz7	[17]	- Zebrafish mRNA injection and overexpression, mesoderm cell migration	no/yes

Supplementary Table 2. optoXR variants generated in this study.

GPCR source sequence	Opto variant	Protein sequence of generated optoXR chimera (Rho/**target receptor residues)**
A0A0B4KHI2.1/ 145-431 Dopamine 1-like receptor 1 isoform E ECO:0000313 EMBL:AGB959 44.1	**Opto Dop1R1 V1**	MKTIIALSYIFCLVFAMYTDIEMNRLGKDSLMNGTEGPNFYVPFSNKTGVVR SPFEAPQYYLAEPWQFSMLAAYMFLLIMLGFPINFLTLYV**IYTERSLRR**ILNYI LLNLAVADLFMVFGGFTTTLYTSLHGYFVFGPTGCNLEGFFATLGGEIALWS LVVLAIERYVVV**KDPLRYGRWVTRR**AIMGVAFTWVMALACAAPPLVGWSR YIPEGMQCSCGIDYYTPHEETNNESFVIYMFVVHFIIPLIVIFFCYG**RLYCYAQ KHVKSIKAVTRPGEVAEKQRYKSIRRPKNQPKKFKVRNLHTHSSPYHVSD HKAA**RMVIIMVIAFLICWLPYAGVAFYIFTHQGSDFGPIFMTIPAFFAKTSAVY NPVIYIMMNKQ**FRDAFKRILTMRNPWCCAQDVGNIHPRNSDRFITDYAAKN VVVMNSGRSSAELEQVSAI**TETSQVAPA
A0A0B4KHI2.1/ 145-431 Dopamine 1-like receptor 1 isoform E ECO:0000313 EMBL:AGB959 44.1	**Opto Dop1R1 V2**	MKTIIALSYIFCLVFAMYTDIEMNRLGKDSLMNGTEGPNFYVPFSNKTGVVR SPFEAPQYYLAEPWQFSMLAAYMFLLIMLGFPINFLTLYVTVQHKKLRTPLN YILLNLAVADLFMVFGGFTTTLYTSLHGYFVFGPTGCNLEGFFATLGGEIAL WSLVVLAIERYVVV**KDPLRYGRWVTRRV**AIMGVAFTWVMALACAAPPLVG WSRYIPEGMQCSCGIDYYTPHEETNNESFVIYMFVVHFIIPLIVIFFCYG**RLYC YAQKHVKSIKAVTRPGEVAEKQRYKSIRRPKNQPKKFKVRNLHTHSSPYH VSDHKAA**RMVIIMVIAFLICWLPYAGVAFYIFTHQGSDFGPIFMTIPAFFAKTS AVYNPVIYIM**FNKEFRDAFKRILTMRNPWCCAQDVGNIHPRNSDRFITDYA AKNVVVMNSGRSSAELEQVSAI**TETSQVAPA
A0A0B4KI18.1/ 125-474 Dopamine 1-like receptor 2 isoform CECO:0000313 EMBL:AGB964 52.1	**Opto Dop1R2 V1**	MKTIIALSYIFCLVFAMYTDIEMNRLGKDSLMNGTEGPNFYVPFSNKTGVVR SPFEAPQYYLAEPWQFSMLAAYMFLLIMLGFPINFLTLYV**VIRERYLHTA**LNY ILLNLAVADLFMVFGGFTTTLYTSLHGYFVFGPTGCNLEGFFATLGGEIALW SLVVLAIERYVVV**TDPFSYPMRMTVK**AIMGVAFTWVMALACAAPPLVGWSR YIPEGMQCSCGIDYYTPHEETNNESFVIYMFVVHFIIPLIVIFFCYG**RIYRAAVI QTRSLKIGTKQVLMASGELQLTLRIHRGGTTRDQQNQVSGGGGGGGGGG GGGGSLSHSHSHSHHHHHNHGGGTTTSTPEEPDDEPLSALHNNGLARHR HMGKNFSLSRKLAKFAKEKKAA**RMVIIMVIAFLICWLPYAGVAFYIFTHQGS DFGPIFMTIPAFFAKTSAVYNPVIYIMMNKQ**FRRAFVRLLCMCCPRKIRRKY QPTMRSKSQCHVAAAMVAASTSFGYHSVNQIDRTLM**TETSQVAPA
A0A0B4KI18.1/ 125-474 Dopamine 1-like receptor 2 isoform C ECO:0000313 EMBL:AGB964 52.1	**Opto Dop1R2 V2**	MKTIIALSYIFCLVFAMYTDIEMNRLGKDSLMNGTEGPNFYVPFSNKTGVVR SPFEAPQYYLAEPWQFSMLAAYMFLLIMLGFPINFLTLYVTVQHKKLRTPLN YILLNLAVADLFMVFGGFTTTLYTSLHGYFVFGPTGCNLEGFFATLGGEIAL WSLVVLAIERYVVV**TDPFSYPMRMTVKR**AIMGVAFTWVMALACAAPPLVG WSRYIPEGMQCSCGIDYYTPHEETNNESFVIYMFVVHFIIPLIVIFFCYG**RIYR AAVIQTRSLKIGTKQVLMASGELQLTLRIHRGGTTRDQQNQVSGGGGGGG GGGGGGGSLSHSHSHSHHHHHNHGGGTTTSTPEEPDDEPLSALHNNGLA RHRHMGKNFSLSRKLAKFAKEKKAA**RMVIIMVIAFLICWLPYAGVAFYIFTH QGSDFGPIFMTIPAFFAKTSAVYNPVIYIM**WSRDFRRAFVRLLCMCCPRKIR RKYQPTMRSKSQCHVAAAMVAASTSFGYHSVNQIDRTLM**TETSQVAPA
Q0IGY0.1/480-738 Leucine-rich repeat-containing G protein-coupled receptor 4 isoform B ECO:0000313 EMBL:ABW094 04.2	**Opto Lgr4 V1**	MKTIIALSYIFCLVFAMYTDIEMNRLGKDSLMNGTEGPNFYVPFSNKTGVVR SPFEAPQYYLAEPWQFSMLAAYMFLLIMLGFPINFLTLYV**RYFYKSRSNVE**L NYILLNLAVADLFMVFGGFTTTLYTSLHGYFVFGPTGCNLEGFFATLGGEIAL WSLVVLAIERYVVV**TRPLKPRDTEKVR**AIMGVAFTWVMALACAAPPLVGWS RYIPEGMQCSCGIDYYTPHEETNNESFVIYMFVVHFIIPLIVIFFCYG**RMLQAI RDSGGGMRSTHSGRENVVA**RMVIIMVIAFLICWLPYAGVAFYIFTHQGSDF GPIFMTIPAFFAKTSAVYNPVIYIMMNKQ**FRQQLRRYCHTLPSCSLVNNETR SQTQTAYESGLSVSLAHLGGGVGGGSGRKRMSHRQMSYL**TETSQVAPA
E1JJ17.1/118-381 Tachykinin-like receptor at 99D isoform B ECO:0000313 EMBL:ACZ950 66.1	**Opto Tk99D V1**	MKTIIALSYIFCLVFAMYTDIEMNRLGKDSLMNGTEGPNFYVPFSNKTGVVR SPFEAPQYYLAEPWQFSMLAAYMFLLIMLGFPINFLTLYV**VMTTKRMRTV**LN YILLNLAVADLFMVFGGFTTTLYTSLHGYFVFGPTGCNLEGFFATLGGEIAL WSLVVLAIERYVVV**IRPLQPRMSKRC**AIMGVAFTWVMALACAAPPLVGWSR YIPEGMQCSCGIDYYTPHEETNNESFVIYMFVVHFIIPLIVIFFCYG**RVGIELW GSKTIGECTPRQVENVRSKRRVV**RMVIIMVIAFLICWLPYAGVAFYIFTHQGS DFGPIFMTIPAFFAKTSAVYNPVIYIMMNKQ**FRYGFKMVFRWCLFVRVGTEP FSRRENLTSRYSCSGSPDHNRIKRNDTQKSILYTCPSSPKSHRISHSGRSA TLRNSLPAESLSSGGSGGGGHRKRLSYQQEMQQRWSGPNSATAVTNSS STANTTQLLS**TETSQVAPA

GPCR source sequence	Opto variant	Protein sequence of generated optoXR chimera (Rho/**target receptor residues**)
E1JGM2.2/107-533 5-hydroxytryptamine (Serotonin) receptor 1B isoform D ECO:0000313 EMBL:ACZ94473.2	**Opto 5-HT1B V1**	MKTIIALSYIFCLVFAMYTDIEMNRLGKDSLMNGTEGPNFYVPFSNKTGVVR<br>SPFEAPQYYLAEPWQFSMLAAYMFLLIMLGFPINFLTLYV**IILERNLQNV**LNYI<br>LLNLAVADLFMVFGGFTTTLYTSLHGYFVFGPTGCNLEGFFATLGGEIALWS<br>LVVLAIERYVVV**TNIDYNNLRTPR**AIMGVAFTWVMALACAAPPLVGWSRYIP<br>EGMQCSCGIDYYTPHEETNNESFVIYMFVVHFIIPLIVIFFCYG**KIYIIARKRIQ**<br>**RRAQKSFNVTLTETDCDSAVRELKKERSKRRAERKRLEAGERTPVDGDG**<br>**TGGQLQRRTRKRMRICFGRNTNTANVYRTSNANEIITLSQQVAHATQHHLI**<br>**ASHLNAITPLAQSIAMGGVGCLTTTTPSEKALSGAGTVAGAVAGGSGSGS**<br>**GEEGAGTEGKNAGVGLGGVLASIANPHQKLAKRRQLLEAKRERKAAR**MV<br>IIMVIAFLICWLPYAGVAFYIFTHQGSDFGPIFMTIPAFFAKTSAVYNPVIYIMM<br>NKQ**FRRAFKRILFGRKAAARARSAKI**TETSQVAPA
Q7KTL9.1/55-319 Adipokinetic hormone receptor isoform C ECO:0000313 EMBL:AAS64647.1	**Opto AkhR V1**	MKTIIALSYIFCLVFAMYTDIEMNRLGKDSLMNGTEGPNFYVPFSNKTGVVR<br>SPFEAPQYYLAEPWQFSMLAAYMFLLIMLGFPINFLTLYV**LTKRRLRGPLR**L<br>NYILLNLAVADLFMVFGGFTTTLYTSLHGYFVFGPTGCNLEGFFATLGGEIAL<br>WSLVVLAIERYVVV**LKPLKRSYNRGR**AIMGVAFTWVMALACAAPPLVGWS<br>RYIPEGMQCSCGIDYYTPHEETNNESFVIYMFVVHFIIPLIVIFFCYG**AIYLEIY**<br>**RKSQRVLKDVIAERFRRSNDDVLSRAKKRTL**RMVIIMVIAFLICWLPYAGVA<br>FYIFTHQGSDFGPIFMTIPAFFAKTSAVYNPVIYIMMNKQ**FRMNNNNPSVNN**<br>**RHTSLSNRLDSSNQLMQKQLTNNSLLNGRGQVMAAAVSATTKLANVVSL**<br>**KGTANGNGSAAAAGTVPITPPLTVTIAPLATDDEANDDSCLSAVTIRCQDQ**<br>**SPIRQKCGDSIELTSVVK**TETSQVAPA
Q9VBP0.2/447-704 Leucine-rich repeat containing G protein-coupled receptor 3 ECO:0000313 EMBL:AAF56490.2	**Opto Lgr3 V1**	MKTIIALSYIFCLVFAMYTDIEMNRLGKDSLMNGTEGPNFYVPFSNKTGVVR<br>SPFEAPQYYLAEPWQFSMLAAYMFLLIMLGFPINFLTLYV**RFIYRDENVA**LNY<br>ILLNLAVADLFMVFGGFTTTLYTSLHGYFVFGPTGCNLEGFFATLGGEIALW<br>SLVVLAIERYVVV**ADPFRGHRSIGNR**AIMGVAFTWVMALACAAPPLVGWSR<br>YIPEGMQCSCGIDYYTPHEETNNESFVIYMFVVHFIIPLIVIFFCYG**ALLISIWR**<br>**TRSATPLTLLDCEFA**RMVIIMVIAFLICWLPYAGVAFYIFTHQGSDFGPIFMTI<br>PAFFAKTSAVYNPVIYIMMNKQ**FRNQIFLRGWKKITSRKRAEAGNGNVATT**<br>**TTGTATGSSQHPDDFTIFAKAAMRCH**TETSQVAPA
Q9VW75.2/80-236 Short neuropeptide F receptor isoform B EMBL:AGB94779.1	**Opto sNPFR V1**	MKTIIALSYIFCLVFAMYTDIEMNRLGKDSLMNGTEGPNFYVPFSNKTGVVR<br>SPFEAPQYYLAEPWQFSMLAAYMFLLIMLGFPINFLTLYV**VLRNRAMQTVTN**<br>**IFITNLALSD**LNYILLNLAVADLFMVFGGFTTTLYTSLHGYFVFGPTGCNLEG<br>FFATLGGEIALWSLVVLAIERYVVV**IYPFHPRMKLST**AIMGVAFTWVMALAC<br>AAPPLVGWSRYIPEGMQCSCGIDYYTPHEETNNESFVIYMFVVHFIIPLIVIFF<br>CYG**WISVKLNQRARAKPGSKSSRREEADRDRKKRTN**RMVIIMVIAFLICWL<br>PYAGVAFYIFTHQGSDFGPIFMTIPAFFAKTSAVYNPVIYIMMNKQFR**YAWL**<br>**NENFRKEFKHVLPCFNPSNNNIINITRGYNRSDRNTCGPRLHHGKGDGGM**<br>**GGGSLDADDQDENGITQETCLPKEKLLIIPREPTYGNGTGAVSPILSGRGIN**<br>**AALVHGGDHQMHQLQPSHHQQVELTRRIRRRTDETDGDYLDSGDEQTVE**<br>**VRFSETPFVSTDNTTGISILETSTSHCQDSDVMVELGEAIGAGGGAELGRRI**<br>**N**TETSQVAPA

Supplementary References

1. Kim, J.-M. *et al.* Light-Driven Activation of β 2 -Adrenergic Receptor Signaling by a Chimeric Rhodopsin Containing the β 2 -Adrenergic Receptor Cytoplasmic Loops †. *Biochemistry* **44**, 2284–2292 (2005).
2. Airan, R. D., Thompson, K. R., Fenno, L. E., Bernstein, H. & Deisseroth, K. Temporally precise in vivo control of intracellular signalling. *Nature* **458**, 1025–1029 (2009).
3. Siuda, E. R. *et al.* Optodynamic simulation of β-adrenergic receptor signalling. *Nat Commun* **6**, 8480 (2015).
4. Siuda, E. R., Al-Hasani, R., McCall, J. G., Bhatti, D. L. & Bruchas, M. R. Chemogenetic and Optogenetic Activation of Gαs Signaling in the Basolateral Amygdala Induces Acute and Social Anxiety-Like States. *Neuropsychopharmacology* **41**, 2011–2023 (2016).
5. Adamsky, A. *et al.* Astrocytic Activation Generates De Novo Neuronal Potentiation and Memory Enhancement. *Cell* **174**, 59-71.e14 (2018).
6. Iwai, Y. *et al.* Transient Astrocytic Gq Signaling Underlies Remote Memory Enhancement. *Front Neural Circuits* **15**, (2021).
7. Gerasimov, E. *et al.* Optogenetic Activation of Astrocytes—Effects on Neuronal Network Function. *International Journal of Molecular Sciences 2021, Vol. 22, Page 9613* **22**, 9613 (2021).
8. Barish, P. A. *et al.* Design and functional evaluation of an optically active μ-opioid receptor. *Eur J Pharmacol* **705**, 42–48 (2013).
9. Siuda, E. R. *et al.* Spatiotemporal Control of Opioid Signaling and Behavior. *Neuron* **86**, 923–935 (2015).
10. Castro, D. C. *et al.* An endogenous opioid circuit determines state-dependent reward consumption. *Nature 2021 598:7882* **598**, 646–651 (2021).
11. Gunaydin, L. A. *et al.* Natural Neural Projection Dynamics Underlying Social Behavior. *Cell* **157**, 1535–1551 (2014).
12. Xu, Y. *et al.* Optogenetic control of chemokine receptor signal and T-cell migration. *Proc Natl Acad Sci U S A* **111**, 6371–6376 (2014).
13. Li, P. *et al.* Optogenetic activation of intracellular adenosine A2A receptor signaling in the hippocampus is sufficient to trigger CREB phosphorylation and impair memory. *Mol Psychiatry* **20**, 1481 (2015).
14. Li, Y. *et al.* Optogenetic Activation of Adenosine A2A Receptor Signaling in the Dorsomedial Striatopallidal Neurons Suppresses Goal-Directed Behavior. *Neuropsychopharmacology* **41**, 1003–1013 (2016).
15. van Wyk, M., Pielecka-Fortuna, J., Löwel, S. & Kleinlogel, S. Restoring the ON Switch in Blind Retinas: Opto-mGluR6, a Next-Generation, Cell-Tailored Optogenetic Tool. *PLoS Biol* **13**, 1–30 (2015).
16. Kralik, J., van Wyk, M., Stocker, N. & Kleinlogel, S. Bipolar cell targeted optogenetic gene therapy restores parallel retinal signaling and high-level vision in the degenerated retina. *Communications Biology 2022 5:1* **5**, 1–15 (2022).

17. Čapek, D. *et al.* Light-activated Frizzled7 reveals a permissive role of non-canonical wnt signaling in mesendoderm cell migration. *Elife* **8**, (2019).
