# Supplementary material for: Optimized design and in vivo application of optogenetically functionalized Drosophila dopamine receptors
Source: Nat Commun. 2023 Dec 19;14:8434. doi: 10.1038/s41467-023-43970-0 (PMC10730509; doi:10.1038/s41467-023-43970-0)
Supplement: Supplementary file 3 — Description of Additional Supplementary Files [file 41467_2023_43970_MOESM3_ESM.pdf]

Description of Additional Supplementary Files

Supplementary Movie Legends:

Supplementary Movie 1: Blue light induced rolling of larvae expressing bPAC in nociceptors (ppk-Gal4>UAS-bPAC)

Supplementary Movie 2: Blue light induced rolling of larvae expressing optoDop1R1V1 in nociceptors (ppk-Gal4>UASoptoDop1R1V1)

Supplementary Movie 3: Blue light induced rolling of larvae expressing optoDop1R1V2 in nociceptors (ppk-Gal4>UASoptoDop1R1V2)

Supplementary Movie 4: Blue light induced rolling of larvae expressing optoDop1R2V2 in nociceptors (ppk-Gal4>UASoptoDop1R2V2)

Supplementary Movie 5: Blue light induced rolling of larvae expressing CsChrimson in nociceptors (ppk-Gal4>UASCsChrimson).

Supplementary Movie 6: cAMP response of blue light induced bPAC activation in the mushroom body soma and medial lobe region (H24-Gal4>UAS-bPAC,UAS-Gflamp1).

Supplementary Movie 7: cAMP response of blue light induced optoDop1R1V2 activation in the mushroom body soma and medial lobe region (H24-Gal4>UAS-optoDop1R1V2,UAS-Gflamp1).

Supplementary Movie 8: Calcium response of blue light induced optoDop1R2V2 activation in the mushroom body soma and medial lobe region (H24-Gal4>UAS-optoDop1R2V2,UAS-GCaMP6s).

Supplementary Movie 9: Locomotion of rotenone-treated larvae expressing optoDop1R1V2 in the endogenous pattern of Dop1R1 using a knock-in Gal4 line (Dop1R1KO-Gal4>UAS-optoDop1R1V2). Larvae were tracked in the dark (magenta tracks) and subsequently upon green light illumination (green tracks).
