# Supplementary material for: Optimized design and in vivo application of optogenetically functionalized Drosophila dopamine receptors
Source: Nat Commun. 2023 Dec 19;14:8434. doi: 10.1038/s41467-023-43970-0 (PMC10730509; doi:10.1038/s41467-023-43970-0)
Supplement: Supplementary file 13 — Reporting Summary [file 41467_2023_43970_MOESM13_ESM.pdf]

nature portfolio

Corresponding author(s): Peter Soba
Last updated by author(s): Oct 31, 2023

Reporting Summary

Nature Portfolio wishes to improve the reproducibility of the work that we publish. This form provides structure for consistency and transparency in reporting. For further information on Nature Portfolio policies, see our Editorial Policies and the Editorial Policy Checklist.

Statistics

For all statistical analyses, confirm that the following items are present in the figure legend, table legend, main text, or Methods section.

n/a	Confirmed	
☐	☒	The exact sample size (*n*) for each experimental group/condition, given as a discrete number and unit of measurement
☐	☒	A statement on whether measurements were taken from distinct samples or whether the same sample was measured repeatedly
☐	☒	The statistical test(s) used AND whether they are one- or two-sided<br>*Only common tests should be described solely by name; describe more complex techniques in the Methods section.*
☐	☒	A description of all covariates tested
☐	☒	A description of any assumptions or corrections, such as tests of normality and adjustment for multiple comparisons
☐	☒	A full description of the statistical parameters including central tendency (e.g. means) or other basic estimates (e.g. regression coefficient) AND variation (e.g. standard deviation) or associated estimates of uncertainty (e.g. confidence intervals)
☐	☒	For null hypothesis testing, the test statistic (e.g. *F*, *t*, *r*) with confidence intervals, effect sizes, degrees of freedom and *P* value noted<br>*Give P values as exact values whenever suitable.*
☒	☐	For Bayesian analysis, information on the choice of priors and Markov chain Monte Carlo settings
☒	☐	For hierarchical and complex designs, identification of the appropriate level for tests and full reporting of outcomes
☒	☐	Estimates of effect sizes (e.g. Cohen's *d*, Pearson's *r*), indicating how they were calculated

Our web collection on statistics for biologists contains articles on many of the points above.

Software and code

Policy information about availability of computer code

Data collection	ZEISS ZEN Blue Software, FIMtrack software (https://github.com/kostasl/ FIMTrack); MicroWin (plate reader); Trikinetics activity monitor;optoPAD (https://github.com/ribeiro-lab/optoPAD-software)
Data analysis	Prism 8.0; Fiji package (ImageJ v2), StackReg (Fiji plugin); TimeSeries AnalyzerV3 (Fiji Plugin); actogramJ (Fiji plugin); FIMTrack software ((https://github.com/kostasl/ FIMTrack); Matlab 2022b

For manuscripts utilizing custom algorithms or software that are central to the research but not yet described in published literature, software must be made available to editors and reviewers. We strongly encourage code deposition in a community repository (e.g. GitHub). See the Nature Portfolio guidelines for submitting code & software for further information.

Data

Policy information about availability of data

All manuscripts must include a data availability statement. This statement should provide the following information, where applicable:

- Accession codes, unique identifiers, or web links for publicly available datasets
- A description of any restrictions on data availability
- For clinical datasets or third party data, please ensure that the statement adheres to our policy

The raw data generated in this study are provided in the Source Data file. The raw imaging data (calcium imaging and immunohistochemistry) generated in this study can be obtained by request from the corresponding author. Source data are provided with this paper.

Research involving human participants, their data, or biological material

Policy information about studies with human participants or human data. See also policy information about sex, gender (identity/presentation), and sexual orientation and race, ethnicity and racism.

Reporting on sex and gender	n/a
Reporting on race, ethnicity, or other socially relevant groupings	n/a
Population characteristics	n/a
Recruitment	n/a
Ethics oversight	n/a

Note that full information on the approval of the study protocol must also be provided in the manuscript.

Field-specific reporting

Please select the one below that is the best fit for your research. If you are not sure, read the appropriate sections before making your selection.

☒ Life sciences ☐ Behavioural & social sciences ☐ Ecological, evolutionary & environmental sciences

For a reference copy of the document with all sections, see nature.com/documents/nr-reporting-summary-flat.pdf

Life sciences study design

All studies must disclose on these points even when the disclosure is negative.

Sample size	Sample-size calculations were not performed as the effect size was not known before the study. Sample sizes for the different experiments in this study were matched to published experiments that used similar methodology and manipulations.<br>GPCR assays: 10.1016/j.neuron.2021.03.013<br>larval learning/preference: 10.1038/s41467-018-03130-1<br>adult learning assays: 10.1371/journal.pbio.3000400<br>activity monitor assays: 10.1177/07487304211032336<br>feeding assays: 10.7554/eLife.43924
Data exclusions	No data were excluded from the analyses except if samples did not meet sufficient quality standards including sufficient cellular expression levels (HEK293 cell assays) or physically damaged samples after dissection. For functional imaging experiments, we excluded samples which showed significant z-drift during imaging. For analysis of larval locomotion we excluded animals that could not be continuously tracked by the tracking software due to loss of signal.
Replication	Representative images were obtained from experiments that were repeated independently at least twice. All other experiments were replicated in biologically independent samples (HEK cells, larval and adult flies) or experiments as indicated by precise n numbers in the figure legends.
Randomization	Staged larvae and adult flies of the genotype of interest were randomly collected from a larger pool. Animals of each group were randomly processed whenever possible.
Blinding	The experimentalist were blinded to the genotype during the experiment and the analysis.

Reporting for specific materials, systems and methods

We require information from authors about some types of materials, experimental systems and methods used in many studies. Here, indicate whether each material, system or method listed is relevant to your study. If you are not sure if a list item applies to your research, read the appropriate section before selecting a response.

Materials & experimental systems

n/a	Involved in the study
☐	☒ Antibodies
☐	☒ Eukaryotic cell lines
☒	☐ Palaeontology and archaeology
☐	☒ Animals and other organisms
☒	☐ Clinical data
☒	☐ Dual use research of concern
☒	☐ Plants

Methods

n/a	Involved in the study
☒	☐ ChIP-seq
☒	☐ Flow cytometry
☒	☐ MRI-based neuroimaging

Antibodies

Antibodies used

mouse anti-Rhodopsin (1D4, Cat #MA1-722, 1:1,000, Thermo Fisher)
rabbit anti-DsRed (Cat #632496, 1:2,000, Takara Bio Inc.)
mouse anti-GFP (1:2,000, Cat #A-11120, Thermo Fisher)
rabbit or guinea pig anti-Discs large (1:30,000 and 1:1.000, Pielage et al. Neuron 2011)
mouse anti-PDF (PDF C7, DSHB, 1:1000)
chicken anti-GFP (Cat #ab13970, Abcam, 1:2000)
2ndary antibodies:
donkey anti-mouse Alexa 488 Cat #715-545-150, Jackson Immunoresearch, 1:300
goat anti-mouse Alexa 546 Cat # A-11030, Thermo Fisher, CA, USA, 1:300
goat anti-mouse Alexa 488 Cat # A-11001, goat anti-rabbit Alexa 594 Cat # A-11012, goat anti-guinea pig Alexa 647 Cat # A-21450, all 1:1,000; donkey anti-mouse Alexa 555 Cat # A-31570, 1:400; goat anti-chicken Alexa 488 Cat # A-11039, 1:200, all Thermo Fisher

Validation

mouse anti-Rhodopsin: company statement "MA1-722 has been successfully used in Western blot, IHC (P), immunocytochemistry and immunoprecipitation procedures. By Western blot, this antibody detects an ~40 kDa protein representing rhodopsin from Sf9 cells expressing the bovine gene. Immunocytochemical staining of rhodopsin in human retinal samples results in staining of both rod and cone outer segments." (https://www.thermofisher.com/antibody/product/Rhodopsin-Antibody-clone-1D4-Monoclonal/MA1-722)
rabbit anti-DsRed: doi.org/10.1073/pnas.1010198107
mouse anti-GFP: This Antibody was verified by Relative expression to ensure that the antibody binds to the antigen stated (https://www.thermofisher.com/antibody/product/GFP-Antibody-clone-3E6-Monoclonal/A-11120)
anti-discs large: doi:10.1016/j.neuron.2011.02.007
mouse anti-PDF: doi: 10.1523/JNEUROSCI.0263-05.2005
chicken anti-GFP: doi: 10.1016/j.neuron.2022.10.034

Eukaryotic cell lines

Policy information about cell lines and Sex and Gender in Research

Cell line source(s)

HEK293T (gift from M. Karsak, ZMNH, University Medical Center Hamburg-Eppendorf), G protein deficient HEK293 cells (deltaG7, Tohoku University, Japan)

Authentication

HEK293T were not further authenticated except for visual inspection,HEK293-deltaG7 cells were functionally tested for the absence of endogenous Gs signaling using the Gsx assay as described in the manuscript

Mycoplasma contamination

not tested for HEK293 cells used in this study

Commonly misidentified lines (See ICLAC register)

no commonly misidentified cell lines were used in the study,

Animals and other research organisms

Policy information about studies involving animals; ARRIVE guidelines recommended for reporting animal research, and Sex and Gender in Research

Laboratory animals

Transgenic Drosophila melanogaster in a w1118 background were used for all experiments. Staged 3rd instar larve (96 h after egg laying) and young adult male flies (1-8d old) were used. The complete list of lines used is shown below.
Dop1R1KO-Gal4
UAS-Dop1R1RNAi
UAS-Dop1R2RNAi
Dop1R2KO-Gal4
201y-Gal4
H24-Gal4
UAS-bPAC
UAS-optoDop1R1V2
UAS-optoDop1R2V2
UAS-optoDop1R1V1

	ppk-Gal4<br>UAS-CsChrimson-GFP<br>UAS-Gflamp1<br>UAS-Gcamp6s<br>MBONg1g2-Gal4<br>Pdf-Gal4<br>MB011B-Gal4<br>2U<br>OK107-Gal4<br>tub-Gal80ts<br>R21B06-splitGal4DBD<br>6xCRE-splitGal4AD<br>UAS-myr::tdTomato<br>UAS-Dop1R1GFP11, UAS-spGFP1-10<br>10xUAS-myr::GFP
Wild animals	No wild animals were used in the study.
Reporting on sex	male and female larvae were used in all experiments, but not analyzed separately due to their asexual state. Only male flies were used for activity monitoring and feeding assays due to the tool-specific proof of principle nature of the experiments.
Field-collected samples	No field collected samples were used in the study.
Ethics oversight	This study did not require ethical approval.

Note that full information on the approval of the study protocol must also be provided in the manuscript.
